# Effect size varies based on calculation method and may affect interpretation of treatment effect: an illustration using randomised clinical trials in osteoarthritis

**DOI:** 10.1186/s42358-024-00358-y

**Published:** 2024-04-22

**Authors:** Thomas J. Schnitzer, Philip G. Conaghan, Francis Berenbaum, Lucy Abraham, Joseph C. Cappelleri, Andrew G. Bushmakin, Lars Viktrup, Ruoyong Yang, Mark T. Brown

**Affiliations:** 1 Northwestern University Feinberg School of Medicine, Chicago, IL USA; 2 Leeds Institute of Rheumatic and Musculoskeletal Medicine, National Institute for Health Research, Leeds Biomedical Research Centre-University of Leeds, Leeds, UK; 3 Sorbonne University, INSERM CRSA, AP-HP Saint-Antoine hospital, Paris, France; 4 Pfizer R&D UK Ltd, Surrey, UK; 5 Pfizer Inc, Groton, CT USA; 6 Eli Lilly and Co, Indianapolis, IN USA; 7 Pfizer Inc, New York, NY USA

**Keywords:** Effect size, Variability, Osteoarthritis, Pain, Randomised controlled trial

## Abstract

**Background:**

To illustrate how (standardised) effect sizes (ES) vary based on calculation method and to provide considerations for improved reporting.

**Methods:**

Data from three trials of tanezumab in subjects with osteoarthritis were analyzed. ES of tanezumab versus comparator for WOMAC Pain (outcome) was defined as least squares difference between means (mixed model for repeated measures analysis) divided by a pooled standard deviation (SD) of outcome scores. Three approaches to computing the SD were evaluated: Baseline (the pooled SD of WOMAC Pain values at baseline [pooled across treatments]); Endpoint (the pooled SD of these values at the time primary endpoints were assessed); and Median (the median pooled SD of these values based on the pooled SDs across available timepoints). Bootstrap analyses were used to compute 95% confidence intervals (CI).

**Results:**

ES (95% CI) of tanezumab 2.5 mg based on Baseline, Endpoint, and Median SDs in one study were − 0.416 (− 0.796, − 0.060), − 0.195 (− 0.371, − 0.028), and − 0.196 (− 0.373, − 0.028), respectively; negative values indicate pain improvement. This pattern of ES differences (largest with Baseline SD, smallest with Endpoint SD, Median SD similar to Endpoint SD) was consistent across all studies and doses of tanezumab.

**Conclusion:**

Differences in ES affect interpretation of treatment effect. Therefore, we advocate clearly reporting individual elements of ES in addition to its overall calculation. This is particularly important when ES estimates are used to determine sample sizes for clinical trials, as larger ES will lead to smaller sample sizes and potentially underpowered studies.

**Trial Registration:**

Clinicaltrials.gov NCT02697773, NCT02709486, and NCT02528188.

**Supplementary Information:**

The online version contains supplementary material available at 10.1186/s42358-024-00358-y.

## Background

Effect sizes (ES) provide information about the magnitude of differences between groups in interventional studies [1, 2]. While treatment differences should be based primarily on the original metric of the outcome (e.g., difference in mean scores between two treatments), the ES when standardised and expressed in standard deviation units can lend further interpretation to the magnitude of effect. Standardised ES are also used to calculate sample sizes for studies and to support comparisons of effects across studies [3, 4]. Comparing standardised ES across interventions or studies, however, must be done with caution as ES may vary depending on study design, outcome measures, and approach to calculation of the standard deviation (SD) [5].

The (standardised) ES metric for a parallel-group clinical trial is defined as the difference in mean scores between two treatments (numerator) divided by the SD of these two treatments (denominator) [6]. However, there are different approaches to defining the SD to be used when computing ES. Therefore, it is of interest to assess the impact of different approaches to defining the SD on ES using data from well-controlled clinical studies. Here, we report results from three phase 3 trials of tanezumab, an antibody to nerve growth factor, in participants with painful knee and hip osteoarthritis. We focus on the ES for the pain response, as it is the outcome most-commonly evaluated.

## Methods

Data were from two phase 3, randomised, double-blind, multicentre, placebo-controlled, parallel-group trials (Study 1: NCT02697773, Study 2: NCT02709486) [7, 8] and one phase 3, randomised, double-blind, multicentre, active-controlled (nonsteroidal anti-inflammatory drugs [NSAIDs]), parallel-group trial (Study 3: NCT02528188) [9]. Trial details have been published previously.

Overall, study treatment (tanezumab, placebo, or NSAID) was received by 696 participants in Study 1, 849 participants in Study 2, and 2996 participants in Study 3 [7–9].

ES calculations of tanezumab *versus* placebo used Western Ontario and McMaster Universities Osteoarthritis Index (WOMAC, ©1996 Nicholas Bellamy; WOMAC® is a registered trademark of Nicholas Bellamy [CDN, EU, USA]) Pain scores at Week 16 (Study 1) or Week 24 (Study 2). ES calculations of tanezumab *versus* NSAIDs used WOMAC Pain scores at Week 16 (Study 3). Mixed model for repeated measures (MMRM) was used to analyse change from baseline on observed data from each study [10]. The model included time (study week), treatment, treatment-by-time interaction, and randomisation stratification variables. Randomisation stratification variables included index joint and highest Kellgren-Lawrence grade, which were treated as fixed effects. Baseline WOMAC Pain scores and baseline diary average pain scores were treated as covariates.

ES were defined as least squares mean difference (from the MMRM model) in each score divided by a pooled SD of the outcome scores. Three different approaches to computing the pooled SD (in the denominator of the ES) were used: the pooled SD of WOMAC Pain values at baseline (combined across treatments); the pooled SD of these values at the time when the primary endpoints were assessed (Week 16 for Studies 1 and 3, Week 24 for Study 2); and the median pooled SD of these values based on the pooled SDs across all available timepoints (baseline, intermediate post-baseline timepoints, and primary timepoint at the end of a trial). Specifically, the median pooled SD was computed as the median of pooled SD from baseline to Week 16 (Studies 1 and 3) or Week 24 (Study 2).

Given there is no convenient closed-form solution to derive standard errors and confidence intervals (CI) for ES statistics, the non-parametric bootstrap approach is recommended to compute a 95% CI for an ES and was applied to individual WOMAC Pain patient data [11]. One thousand data sets were sampled from individual patient WOMAC Pain data. The bootstrap was done at the patient-level; if a patient was selected, all WOMAC Pain data (at all visits) for this patient were selected. The bootstrap was performed with replacement, using the same number of patients as the original sample. The bootstrap sample data set was used to compute pooled SDs. For each study, each treatment comparison, and each approach to calculate SD (baseline, endpoint, and median), the 95% CI (2.5% percentile, 97.5% percentile) of the ES were reported.

## Results

### Standard deviations

The pooled baseline SDs were the smallest and the pooled SDs at the time when the primary endpoints were assessed were the largest for the WOMAC Pain endpoint in all studies. The SDs for the median of pooled SD were similar to those determined at the primary endpoint (Table 1). SDs across studies were comparable (Table 1).

**Table 1:** Standard deviations used to calculate the ES

Standard deviation method	Study 1	Study 2	Study 3
WOMAC Pain subscale score			
Pooled SD at baseline	1.19	0.92	1.11
Pooled SD at the time when the primary endpoints were assessed	2.66	2.19	2.42
Median pooled SD from baseline to the time when the primary endpoints were assessed	2.56	2.05	2.25

ES: effect size: SD: standard deviation; WOMAC: Western Ontario and McMasters Universities Osteoarthritis Index

### Effect sizes

Based on the bootstrapping method, the mean (95% CI) ES of tanezumab 2.5 mg on pain were − 0.416 (− 0.796, − 0.060) *versus* placebo in Study 1 when pooled baseline SDs were used; −0.195 (− 0.371, − 0.028) when pooled SDs at the time primary endpoints were assessed; and − 0.196 (− 0.373, − 0.028) when the median of pooled SDs from baseline to the time when the primary endpoints were assessed (Table 2). In Study 2, the corresponding ES of tanezumab 2.5 mg on pain *versus* placebo were − 0.547 (− 0.900, − 0.208), − 0.250 (− 0.403, − 0.095), and − 0.256 (− 0.414, − 0.098), respectively (Table 2). In Study 3, the ES of tanezumab 2.5 mg on pain *versus* NSAID were − 0.167 (− 0.324, 0.001), − 0.084 (− 0.163, 0.001), and − 0.085 (− 0.165, 0.001), respectively (Table 2). Similar patterns of differences in ES (based on the SD calculation method) were obtained for higher doses of tanezumab (Table 2).

**Table 2:** ES of tanezumab on pain as measured by WOMAC Pain score based on bootstrap samples

ES, mean (95% CI)	Study 1 (vs. placebo)		Study 2 (vs. placebo)		Study 3 (vs. NSAID)	
	Tanezumab 2.5 mg (*N* = 231)	Tanezumab 2.5/5 mg (*N* = 233)	Tanezumab 2.5 mg (*N* = 283)	Tanezumab 5 mg (*N* = 284)	Tanezumab 2.5 mg (*N* = 1002)	Tanezumab 5 mg (*N* = 998)
Pooled baseline SD	−0.416 (− 0.796, − 0.060)	−0.569 (− 0.952, − 0.179)	−0.547 (− 0.900, − 0.208)	−0.691 (− 1.06, − 0.352)	−0.167 (− 0.324, 0.001)	−0.255 (− 0.454, − 0.062)
Pooled SD at the time when the primary endpoints were assessed*	−0.195 (− 0.371, − 0.028)	−0.267 (− 0.447, − 0.085)	−0.250 (− 0.403, − 0.095)	−0.316 (− 0.482, − 0.163)	−0.084 (− 0.163, 0.001)	−0.128 (− 0.228, − 0.032)
Median of pooled SD from baseline to the time when the primary endpoints were assessed*	−0.196 (− 0.373, − 0.028)	−0.268 (− 0.444, − 0.085)	−0.256 (− 0.414, − 0.098)	−0.323 (− 0.492, − 0.165)	−0.085 (− 0.165, 0.001)	−0.129 (− 0.231, − 0.032)

CI: confidence interval; ES: effect size; NSAID: nonsteroidal anti-inflammatory drug; SD: standard deviation; WOMAC: Western Ontario and McMasters Universities Osteoarthritis Index

* Week 16 for Studies 1 and 3, Week 24 for Study 2

## Discussion

Different approaches to calculating pooled SD affect the magnitude of ES, which in turns affects interpretation of treatment effect and complicates comparisons across different studies. Our results showed that ES derived from pooled SDs, at the time when the primary endpoints were assessed and from the median pooled SDs from baseline to the time when the primary endpoints were assessed, were similar for all endpoint comparisons in all three studies. However, ES derived from pooled SDs at baseline were larger than the ES derived from the other two SDs for all endpoint comparisons in all studies.

All three approaches to calculate SD attempt to estimate “true” variability of the measured outcome in the sample. Use of only baseline data for the SD represents natural variability in the sample, which is not affected by introduction of a treatment (assuming the outcome was not an entry criterion). SDs based at the primary endpoint are calculated by pooling data by treatment and, thus, effectively exclude the treatment effect (as the pooled SD is based on a weighted average of each treatment’s SD of scores rather than an overall SD of scores lump summed as one grouping from both treatment groups; see Supplementary Text 1 for more detail). Using median SD from the set of pooled SDs represents an attempt to use a representative value of variability.

For patient-reported outcome studies, ES using baseline SD or SD of individual changes are typically used for within group pre- *versus* post-intervention comparisons. For ES comparison between treatment groups, the pooled SD from scores of the treatment groups at baseline, pooled SD from scores of the treatment groups at time of post-treatment assessment, or pooled SD from scores of individual changes (when mean change from baseline is the outcome) have been applied [5, 12]. For a clinical trial where the outcome measures also serve as inclusion/exclusion criteria, the population studied at baseline will not represent an unbiased sample. Indeed, the goal of entry criteria is to define a homogeneous population, and it is expected that baseline SD values will be smaller. Furthermore, since response to treatment varies across individuals, SDs based on data after treatment initiation will likely be confounded by effects of treatment and time. Therefore, pooled SD at baseline and pooled SD at post-treatment assessment could be different, which would lead to the differences in ES presented here.

Different factors have been shown to have an impact on the ES of scores in randomised controlled trials [13]. However, our analyses have shown the methods used to calculate the SD directly affect the calculated ES. ES derived from baseline SD tend to be more optimistic (i.e., larger) than ES derived from SD post-treatment. It is noteworthy that the commonly used Cohen thresholds—in which an ES < 0.20 indicates trivial effect; while small, moderate, large, or very large effect is represented by ES of ≥ 0.20 and < 0.50, ≥ 0.50 and < 0.80, ≥ 0.80 and < 1.30, or ≥ 1.30, respectively [14]—were developed for use in the social sciences and are based on Cohen’s *d* when, gauging the magnitude of the difference in means between treatment groups, the pooled standard deviation of scores (pooled across treatments) are based on the same time as when the means are assessed. In contrast, the Cochrane Handbook recommends using the SD from the pooled outcome data (known as Hedges’ g). Thus, when describing the magnitude of an ES, and particularly when comparing across different studies and interventions, it is essential to describe how the SD was determined in order to make appropriate comparisons. This is of even greater importance when using ES estimates to determine sample sizes for clinical trials, as larger effect sizes will lead to smaller sample sizes for equivalent power and may lead to underpowered studies.

Generally, if an outcome scale was not used as part of a study’s entry criteria, we recommend using baseline SD for calculations of ES in longitudinal studies since those SDs are not affected by treatments. If an outcome scale was part of the entry criteria or was highly similar to a measurement used as part of the study’s entry criteria, then baseline SD will be artificially attenuated. In this case, we recommend using the largest pooled post-baseline SD measured at different time points across two (or more) treatment arms since it would lead to the smallest (most conservative) ES. However, ES based on pooled SDs at end of study can also be reported in sensitivity analyses.

## Conclusion

Standardisation of the method used to determine SD would allow researchers to more accurately compare the magnitude of treatment effects across studies, including when different measures are being used to assess the same concept of interest. In the absence of such standardisation, we advocate for reporting, in addition to ES, information about how the individual elements (e.g., means, SDs) were defined/calculated.

## Electronic supplementary material

Below is the link to the electronic supplementary material.

Supplementary Material 1: Text explanation of the term “Pooled SD”

## Data Availability

Upon request, and subject to review, Pfizer will provide the data that support the findings of this study. Subject to certain criteria, conditions and exceptions, Pfizer may also provide access to the related individual de-identified participant data. See https://www.pfizer.com/science/clinical-trials/trial-data-and-results for more information.

## Abbreviations

CI: confidence interval
ES: effect size
MMRM: mixed model for repeated measures
NSAIDS: nonsteroidal anti-inflammatory drugs
SD: standard deviation
WOMAC: Western Ontario and McMaster Universities Osteoarthritis Index
