# Supplementary material for: Effect size varies based on calculation method and may affect interpretation of treatment effect: an illustration using randomised clinical trials in osteoarthritis
Source: Adv Rheumatol. 2024 Apr 22;64:31. doi: 10.1186/s42358-024-00358-y (PMC13488644; doi:10.1186/s42358-024-00358-y)
Supplement: Supplementary file 1 [file 42358_2024_358_MOESM1_ESM.docx]

**Supplementary Text 1.**

“Pooled SD” means that the SD is based on a weighted average of each treatment’s SD of scores.

This approach effectively excludes treatment effect from the variability estimation. If pooled SD is not used, then treatment effect will be included in the SD calculation. As an example, let’s consider two hypothetical treatment arms (placebo and active) and an outcome of pain intensity on a numeric rating scale of 0–10. The active treatment arm has a mean pain score at study endpoint of 2 points with a SD of 1, while the mean pain score for placebo is 6 points with an SD of 1. The pooled SD using data from both arms, therefore, will also be 1. If we calculate an overall SD (without pooling), however, then the SD value will account for differences in means and will obviously be larger in magnitude (about 2.2 in our example assuming normality of the pain scores distributions). Using the pooled SD, the ES calculation would be (2 – 6)/1. In contrast, using the overall SD, the ES calculation would be (2 – 6)/2.2.
